# An Epidemic of Incompetence: A Critical Review of Addictions Curriculum in Canadian Residency Programs

**DOI:** 10.15694/mep.2019.000003.1

**Published:** 2019-01-03

**Authors:** Anees Bahji

**Affiliations:** 1 Queen's University

**Keywords:** Humans, United States, Canada, Education, Medical, Curriculum, Analgesics, Opioid, Behavior, Addictive, Substance-Related Disorders, Psychiatry

## Abstract

This article was migrated. The article was marked as recommended.

In Canada and the United States, the rising number of apparent opioid-related deaths have given to the aptly-named opioid epidemic. Despite the criticism physicians have received for their role in opioid overprescribing, physicians may very well be in the position to vanquish the opioid epidemic. While the importance of the importance of Addictions training in psychiatry and other disciplines has been recognized in Canada at a national level, training resources are scarce and difficult to implement, even when delivered in online formats. Many have speculated that the delivery of high-quality Addictions training has been hampered by multiple roadblocks endemic to the Canadian medical education system, particularly stigma towards individuals with substance use disorders. In navigating the winds of change in the Competency-Based Medical Education (CBME) era, it remains unclear how Addictions will be embraced. To date, there are no defined addictions competencies in the Canadian CBME infrastructure, despite the critical findings of the Association of Faculties of Medicine report in 2017, which was generated in response to the opioid epidemic. Despite these challenges, those who struggle with addiction can lead full, happy, productive lives if they have the right resources. With time, we can only hope that the increasing visibility of addiction will translate to improved training and curricula for the next generation of physicians.

## Background

In Canada and the United States, the rising number of apparent opioid-related deaths have given to the aptly-named opioid epidemic ( Government of Ontario, 2016; Health Canada, 2018). The significant abuse potential of opioids and the ever-increasing prevalence of high potency opioids, such as heroin, fentanyl and its derivatives, have been named as key drivers of the opioid epidemic ( Bahji and Bajaj, 2018). However, with growing certainty, research has indicated that inappropriate prescribing of opioids in vulnerable individuals may underlie a large proportion of opioid overdose fatalities ( Butler et al., 2016; Health Quality Ontario, 2018). As a result of this finding, physicians have come under fire from multiple sources for their contributions to the current opioid crisis.

## Addictions Training in Psychiatry

Despite the criticism we have received, physicians may very well be in the position to vanquish the opioid epidemic. Fortunately, the evidence is quite clear that problematic substance use is a health condition that can be managed and treated effectively (U.S. Department of Health and Human Services (HHS), Office of the Surgeon General, 2012). However, as the delivery of evidence-based care in Addictions demands a specific and exquisitely-complex skill set, many have turned to psychiatry for guidance ( American Academy of Addiction Psychiatry, 2018; Shorter and Dermatis, 2012). During psychiatric training, we gain an unprecedented amount of exposure to a highly stigmatized patient population, which encourages the development of nonjudgmental attitudes towards individuals from all walks of life. We also gain the ability to screen and diagnose a selection of complex mental disorders and are able to utilize these skills to support a population of patients that are perhaps the most challenging in all of medicine. As well, our proficiency with biopsychosocial formulation, our knowledge of complex pharmacotherapy, our expertise with multiple modalities of psychotherapy, and our burgeoning utilization of evidence-based psychiatry equip us with a full armamentarium for individuals with complex, severe, and persistent forms of mental illness, including - but not limited to - substance use disorders. In some ways, the practice of psychiatry embodies the belief that the opposite of Addiction is not abstinence, but connection ( Hari, 2015).

Fortunately, the importance of Addictions training in psychiatry has been recognized in Canada at a national level. In 2015, the Canadian Psychiatric Association (CPA) released a two-part position paper, which specifically outlines training in Addictions ( Crockford et al., 2015; Fleury et al., 2015). In the first part (Supplementary Table 1), the authors provided an overview of training, defining six domains of competence in Addictions, and made six recommendations for how these ought to implemented in Canadian psychiatric residency programs. In the second part (Supplementary Table 2), the authors updated the Addictions training objectives and provided detailed recommendations for the clinical and seminar content in the Addictions curriculum. Overall, the authors proposed stage-specific competencies in Addictions, organized into knowledge and skills domains across the training spectrum.

## Barriers to Providing Addictions Training

Despite the dissemination of the CPA guidelines, the potential of psychiatry to spearhead the response to the opioid epidemic has not yet been realized. In 2017, the Association of Faculties of Medicine of Canada reviewed the accreditation standards for best practices of teaching in Addictions (as well as opioid prescribing and chronic pain management) across all Canadian medical schools and residency programs. Expectedly, they found significant heterogeneity, identifying diverse methodologies of teaching, curricula, and definitions of competency and divergent pedagogies. In the postgraduate medical education category, none of the seventeen Canadian psychiatry programs were deemed to be offering best practice standards in Addictions education ( The Association of Faculties of Medicine of Canada, 2017). Thus, while psychiatrists play a key role in the treatment of patients with Addictive disorders, the next generation of psychiatrists is clearly not receiving training in Addictions.

Not surprisingly, physician surveys have identified that the majority report a lack of confidence in working in the area of Addictions (O’Gara et al., 2005). Logically, it would follow that Addictions training should be clinically grounded to alleviate these perceived low levels of reported clinical training and the resulting lack of confidence. Unfortunately, while many have suggested that medical students, residents, and allied health practitioners (such as nursing students and practicing nurses) be provided with more resources so that they can acquire the key concepts and skills in Addiction, resources are scarce and difficult to implement, even when delivered in online formats.

Some have speculated that the delivery of high-quality Addictions training in psychiatry has been hampered by multiple roadblocks endemic to the Canadian medical education system ( van Boekel et al., 2013). Although illness of any kind need not define an individual, mental and physical illnesses are often regarded differently, and nowhere is that more apparent than in Addictions. A recent systematic review found that negative attitudes of physicians towards patients with substance use disorders were not only highly prevalent, but also a key contributor to suboptimal health care delivery for these patients ( van Boekel et al., 2013). Thus, the undue propagation of stigma towards Addictions is a likely culprit.

However, the inherent characteristics of Addictions also contribute to the difficulties in providing high-level training to the next generation of physicians. For example, the inherent multidisciplinary and interprofessional demands of Addictions mean that it is impossible to assign a single discipline or role to accept the full responsibility of its management. Yet, society has encouraged Addictions scapegoating in the wake of the opioid epidemic, with an expectation that at a certain point, someone (or something) will be held accountable for the ‘problem’. Furthermore, the complexity of Addictions is often difficult to align with the conventional model of medical education, which has often led to the inappropriate distillation to “substance use disorders” or “screening for red flags”.

These issues may be propagated further by the current infrastructure of subspecialty training in Canada and the United States, where there are two competing medical disciplines dedicated to Addictions - Addiction Medicine and Addiction Psychiatry. In the United States, the former receives recognition from the Addiction Medicine Foundation and the American Society of Addiction, while the latter is governed by the Accreditation Council for Graduate Medical Education and the American Academy of Addiction Psychiatry. In Canada, Addiction Psychiatry is not yet a Royal College-recognized discipline in the way that Geriatric, Child, or Forensic Psychiatry are. While it is important to create training pathways in Addictions, this divergence suggests there is a substantive distinction between Addiction Medicine physicians and Addiction Psychiatrists that does not exist. Leading specialists in both disciplines agree on the definition of Addiction and that its treatment is both an art and a science, which requires a multidisciplinary approach. Despite this extensive accord, practitioners of each draw sharp distinctions between Addiction Medicine and Addiction Psychiatry to serve historical, economic, and professional interests, revealing the importance to both disciplines of recognition from their distinctive colleges, and thus, jurisdiction over the medical treatment of addiction.

Despite this, the findings of some key ethnographic research studies have been particularly staggering. For example, in Philadelphian studies of patients who interviewed about their experience with the health care system by outreach nurses, one man said “I don’t want to live under the bridge, but I can’t stop. I don’t know how to access treatment..I don’t have internet..I don’t have phone” ( Carlson et al., 2009). These findings suggest that providers may be unfamiliar with the sociodemographic factors that patients inevitably return to upon discharge from hospital, which exponentially increases the odds of recidivism. This, then, informs the so-called vicious cycle of addiction ( Mazhnaya et al., 2016).

Additional barriers lie in bureaucracy around access to Addictions treatments. For example, while physicians must receive extensive training and certification before they are able to prescribe opioid agonist therapies (like methadone and buprenorphine), similar requirements are not currently in place for the prescription of other controlled substances, such as psychostimulants, benzodiazepines, and even other opioids (like fentanyl). This discrepancy discourages training in and pursuit of clinical work in Addictions, which is already heavily steeped in stigma from multiple levels.

Unfortunately, internalized stigma towards Addictions exists within psychiatry, too. Historically, the relationship between psychiatry and addictions has been complicated by divergent ideologies and attitudinal beliefs and has resisted the inclusion of Addictions in the Diagnostic and Statistical Manual of Mental Disorders ( American Psychiatric Association, 2013). Although there is a gradual move towards integrated care, the remnants of a longstanding tradition that Addiction is not truly a psychiatric illness persist, even today ( Avery et al., 2017; Gertler and Ferneau, 1974; Kochański and Cechnicki, 2017). For example, we still refer to Addictive disorders as ‘concurrent’, which implies that the substance use is a separate - or non-psychiatric - entity ( Danda, 2012). While psychiatry is one - if not the only - discipline that aims to formally incorporate Addictions into its training curriculum ( Royal College of Physicians and Surgeons of Canada, 2015), few psychiatrics manage patients with Addictive disorders ( Kochański and Cechnicki, 2017). Thus, the division between Addictions and psychiatry is ever present.

## The Relationship between CBME and Addictions

In navigating the winds of change in the Competency-Based Medical Education (CBME) era, it remains unclear how Addictions will be embraced ( Khenti et al., 2017). To date, there are no defined Addictions competencies in the Canadian CBME infrastructure, despite the critical findings of the AFMC report in 2017 ( The Association of Faculties of Medicine of Canada, 2017). With that in mind, the multidisciplinary and inherently complex nature of Addictions makes it difficult to generate an entrustable professional activity or discipline-specific tasks. However, simplified Addictions EPAs, such as ‘management of substance use disorders’ and ‘management of substance intoxication and withdrawal’, have been created in other countries (The Royal Australiand and New Zealand College of Psychiatrists, 2012: 3). This suggests that there are possibilities of applying the CBME model to Addictions, even though it may not efficiently address the full breadth of Addictions. If created, this may staunch ongoing criticism that the medical profession has divorced competence from promotion ( Prost, 2018).

Surveys of trainees in Addictions Fellowship Programs have identified preferred curricula. For example, reflection techniques were endorsed as extremely valuable by students, especially in the development of professional attitudes that will help clinicians effectively engage and provide appropriate care for individuals suffering from Addictive disorders. These reflective practices could be used more extensively in psychiatric training in order to build and establish reflexive self-awareness as a core professional competence, which is essential to working effectively in clinical practice, especially in the most demanding contexts ( Ballon and Skinner, 2008).

## The Future of Addictions: Interdisciplinary Collaboration

As the management of Addictions is a multidisciplinary endeavor, provision of care for individuals with Addictive should be a shared responsibility between physicians and health care providers of diverse backgrounds. Several organizations have made concerted efforts to promote addictions stewardship and education among multiple specialties ( Lagisetty et al., 2017; MetaPHI, 2017; Ng, 2018). Mentoring, Education, and Clinical Tools for Addiction: Primary Care-Hospital Integration (META:PHI) is one such example. META:PHI is a provincial initiative based out of Women’s Hospital in Toronto and is mandated to support health care providers in treating individuals struggling with addiction. They provide free open-access information about models of addiction care, clinical addiction tools for health care providers, resources for patients, and information about rapid access addiction medicine (RAAM) clinics across the province.

As innovative approaches to Addictions are desperately needed, interdisciplinary collaboration will be key. Collaboration with public health specialists, epidemiologists, and community services may very well be the missing piece in addressing the educational deficiencies in Addictions. By exploring patterns of Addictions-related health service use at population and community levels, this will enable our medical education system target and address its own deficiencies. Collaboration between provincial and local health organizations can address challenges in operationalizing Addictions services, such as RAAM clinics. Collaboration with knowledge translation agencies, like the Canadian Society of Addiction Medicine, will be helpful in disseminating the evidence-based in Addictions and in unifying our approaches to national issues, such as position papers regarding the implications of the recent legalization of cannabis ( Fischer et al., 2017; Tibbo et al., 2018). Collaboration between hospitals and academic institutions will identify the most cost-effective interventions to maximize finite resources, and can address the local impacts of Addiction; for example, Addictions training on the psychiatric management of bacterial endocarditis related to injection drug use has enabled local cardiac surgeons to be more aware of addictions and understand addictions a bit better ( Yanagawa et al., 2018).

## Reducing Harm by Reducing Stigma

Ideally, we should respond to Addiction in a manner akin to cancer, where recovery is celebrated and even encouraged, and the individual is not blamed for their current state. Admittedly, individuals with Addictive disorders often cut an intimidating figure - with a formidable combination of psychosocial shortcomings, medical frailties, and bold personalities, and often, this rationalizes stigma. But ultimately, they are still regular people, as are addicted women who become pregnant - they have the same right to a healthy pregnancy as any other woman and could do without incessant reminders that they are morally bereft. But physicians are people too, and we must be kind to each other if we are to move forward. We increasingly understand that removing the stigma associated with Addictions is a timely solution as the humanity we all share is more important the illness we may not. The American Society of Addiction Medicine has posted extensively about stigma on their website, which is informed by the findings of international research, such as the decriminalization of illicit drugs in Portugal ( Laqueur, 2015). Such harm reduction programs have unequivocally demonstrated that stigma can be reduced by focusing on rehabilitation, which Canada has emulated with the recent legalization of cannabis. Harm reduction approaches like these respects the frailties of the human condition and acknowledges that unwanted outcomes still happen even with best intentions and best services. However, harm reduction is the first step on the continuum of Addictions recovery, which also includes prevention, treatment, post-treatment, reintegration into society, and ways to inspire others.

There is a tremendous need to implode the myths of Addiction - to put a face on it and to show people that having an Addiction does not have to lead to a painful and oblique life. In recent years, there is a move to recovery capital - the lifelong journey of self-treatment and discipline that guides many Addictions programs. However, the idea remains controversial as managing a severe mental illness is more complicated than simply avoiding certain behaviours. In the words of Elyn Saks, “approaches include medication (usually), therapy (often), a measure of good luck (always) - and, most of all, the inner strength to manage one’s demons, if not banish them. That strength can come from any number of places: love, forgiveness, faith in God, a lifelong friendship” ( Saks, 2014).

## Conclusion

Despite these challenges, those who struggle with addiction can lead full, happy, productive lives if they have the right resources. With time, we can only hope that the increasing visibility of addiction will translate to improved training and curricula for the next generation of physicians.

## Take Home Messages

• In Canada and the United States, the rising number of apparent opioid-related deaths have given to the aptly-named opioid epidemic; physicians have come under fire from multiple sources for their varying contributions to the current opioid crisis.
• Delivery of optimal addictions education and training for the next generation of physicians is hampered by multiple barriers, including stigmatized views towards patients with substance use disorders, the inherently complex demands of patients who struggle with addiction, current infrastructure of subspecialty training which lacks clearly defined roles for training in addictions, and administrative roadblocks to the provision of evidence-based addictions therapies.
• In navigating the winds of change in the Competency-Based Medical Education (CBME) era, it remains unclear how Addictions will be embraced; to date, there are no defined Addictions competencies in the Canadian CBME infrastructure.
• As the management of Addictions is a multidisciplinary endeavor, provision of care for individuals with Addictive should be a shared responsibility between physicians and health care providers of diverse backgrounds.
• Ideally, we should respond to Addiction in a manner akin to cancer, where recovery is celebrated and even encouraged, and the individual is not blamed for their current state.
• Despite these challenges, those who struggle with addiction can lead full, happy, productive lives if they have the right resources.

## Notes On Contributors

Dr. Anees Bahji is a PGY4 resident in the Department of Psychiatry at Queen’s University. He is also enrolled in the Clinician Investigator Program (CIP) at Queen’s University, where he is pursuing a concurrent MSc in Epidemiology in the Department of Public Health Sciences. His primary clinical and research interests are evolving, but he has a special interest in addictions, mental health stigma, concurrent disorders, and psychiatric epidemiology.

Anees Bahji: https://orcid.org/0000-0002-3490-314X
