# Supplementary material for: An Epidemic of Incompetence: A Critical Review of Addictions Curriculum in Canadian Residency Programs
Source: MedEdPublish (2016). 2019 Jan 3;8:3. [Version 1] doi: 10.15694/mep.2019.000003.1 (PMC10712642; doi:10.15694/mep.2019.000003.1)
Supplement: Peer review history

# Reviewer report: Reviewer response for version 1

**DOI:** 10.21956/mep.19743.r29601

**Published:** 2019-01-04

**Authors:** Balakrishnan Nair

**Affiliations:** 1 Centre for Medical Professional Development and University of Newcastle

This review has been migrated. The reviewer awarded 3 stars out of 5 As the author points out , addiction is a common medical issue and does not get the mileage it deserves in undergraduate and post graduate training programs. In the era of Competency Based Education, this is vital. So the article is very timely. I think the title can be refined if the “an epidemic of Incompetence’ can be removed. I think these words do not contribute anything and could be misleading.Our current attitude carries stigma to people with addiction and it is an educational issue for health care professionals. Addiction is an educational issue for patients too and there are many good examples. The patients should be educated and not stigmatised or penalised. The article discusses the current status in Canada and USA. It will be same across the globe and some discussions would have been worthwhile It would have been easier to comprehend , if the author described the psychiatric training and undergraduate training separately. As discussed, this Is an area where Inter professional approach could be the way forward

Reviewer Expertise:

NA

No decision status is available

# Reviewer report: Reviewer response for version 1

**DOI:** 10.21956/mep.19743.r29602

**Published:** 2019-01-03

**Authors:** Trevor Gibbs

**Affiliations:** 1 AMEE

This review has been migrated. The reviewer awarded 4 stars out of 5 Thank you for asking me to review this paper. I felt that it was a very well written paper that certainly brought me up to speed with the present addiction issues in medical education- a very important area. I would not have placed this in the "review of the literature" category, because I felt that there was a lot of personal views brought out here; that did not spoil the paper however. As a retired UK General Practitioner, exposed to rather dreadful situations with opioid addicts and having very little support in how I look after them, I can feel the frustration within the author. I particularly liked the inter-professional approach the author suggested and certainly in the UK, that in itself has brought about improvement in care.I felt that there is still confusion as to if this subject is an undergraduate or postgraduate one; personally and rather sadly, everyone who graduates will come across addiction in some form or another and I would advocate for an insertion of addiction and all that it entails into an undergraduate programme- at least to some slightly more than basic level.As someone who teaches in the Russian Federation and Mainland China I have never seen any mention of addiction teaching at either undergraduate or postgraduate levels, despite the enormity of the problem in their respective communities. I would therefore recommend this paper to all undergraduate curriculum managers / organisers / developers as well as those involved in postgraduate psychiatry training.

Reviewer Expertise:

NA

No decision status is available

# Reviewer report: Reviewer response for version 1

**DOI:** 10.21956/mep.19743.r29603

**Published:** 2019-01-03

**Authors:** Ken Masters

**Affiliations:** 1 Sultan Qaboos University

This review has been migrated. The reviewer awarded 3 stars out of 5 A useful and insightful paper. It indicates a problem from which many medical disciplines suffer – the time delay between research, Guidelines, and application by practitioners. In this case, however, the practitioners are the teachers, and there are added complexities noted by the author, such as the problems caused by having different accreditation bodies, and old and entrenched ideas.Perhaps the consolation at this stage is that barely 3 years have passed since the issuing of the Guidelines – in medical practice, some research has found that the time taken to practise is around 10 years. The message from that is that one must have patience, although the frustration in the meantime is well noted. One can only hope that readers of this paper will be influenced in their practice.To be fair also, the author writes: “In the second part (Supplementary Table 2), the authors updated the Addictions training objectives and provided detailed recommendations for the clinical and seminar content in the Addictions curriculum.” While the Table does give information regarding the curriculum, it is not very detailed. For an institution to take this information and develop (or re-structure) an entire curriculum, and then implement it, will take time. (There may be more details in the references cited by the author, but they are not really present in the Tables, certainly not to the point where a curriculum can be fully developed and rolled out with any ease). It is possible that the author really does need to have more patience.I felt also, that the second part of the paper could have benefited from a more direct link to educational practices, as the paper does tend to veer towards medical rather than medical education. Nevertheless, overall, it is a useful paper giving insight into a most troubling situation.

Reviewer Expertise:

NA

No decision status is available
